# Supplementary material for: Characterization of Rare Himalayan Balsam (Impatiens glandulifera Royle) Honey from Croatia
Source: Foods. 2022 Sep 29;11(19):3025. doi: 10.3390/foods11193025 (PMC9563292; doi:10.3390/foods11193025)
Supplement: Supplementary file 1 [file foods-11-03025-s001.zip › foods-1911827-supplementary.pdf]

Table S1. Detailed insight into pollen spectrum of Himalayan balsam honey samples (n=10; HBH1–HBH10) with regard to predominant (>45%), secondary (16%–45%), important minor (3%–15%), and minor (<3%) pollen of determined plant taxa

**Sample**	**Predominant pollen (>45%)**	**Secondary pollen (16-45%)**	**Important minor pollen (3-15%)**	**Minor Pollen (<3%)**
HBH 1	*I. glandulifera* 75%	-	Fabaceae 6%, Brassicaceae 6%, *Salix* spp. 4%	*Frangula alnus* 2%, *Tilia* spp. 2%, Asteraceae (*Solidago* form) 1%, Asteraceae (*Helinathus* form) 1%
HBH 2	*I. glandulifera* 61%	*Solidago* spp. 27%	Fabaceae 6%, *Fagopyrum esculentum* 4%	*Hedera helix* 1%, undetermined 1%
HBH 3	*I. glandulifera* 64%	-	*Solidago* spp. 15%, Fabaceae 9%, Brassicaceae 9%, undetermined 4%	*Fagopyrum esculentum* 2%
HBH 4	*I. glandulifera* 66%	Brassicaceae 21%	Asteraceae (*Solidago* form) 4%, *Prunus* spp. 3%	undetermined 2%, Fabaceae 1%, Asteraceae (*Helinathus* form) 1%, *Salix* spp. 1%, *Frangula alnus* 1%
HBH 5	*I. glandulifera* 83%	-	*Solidago* spp. 8%	Asteraceae (*Helinathus* form) 2%, Brassicaceae 2%, undetermined 2%, Apiaceae 1%, Rosaceae 1%, Fabaceae 1%
HBH 6	*I. glandulifera* 59%	Fabaceae 16%	undetermined 5%, Brassicaceae 4%, *Frangula alnus* 4%, Asteraceae 4%, *Fagopyrum esculentum* 4%, *Trifolium pratense* 4%	-
HBH 7	*I. glandulifera* 80%	-	Brassicaceae 5%, *Solidago* spp. 5%, undetermined 4%	Rosaceae 2%, Fabaceae 2%, *Salix* spp. 1%, Asteraceae (*Helinathus* form) 1%
HBH 8	*I. glandulifera* 62%	-	Brassicaceae 12%, Solidago spp. 7%, undetermined 4%, Rosaceae 3%	Asteraceae (*Helinathus* form) 2%, Salix spp. 2%, Fabaceae 2%, Asteraceae (*Taraxacum* form) 1%, Asteraceae (*Achilea* form) 1%, *Centaurea* spp. 1%, *Galium* spp. 1%, Lamiaceae 1%, *Viola tricolor* 1%
HBH 9	*I. glandulifera* 75%	-	*Castanea sativa* 9%, *Solidago* spp. 7%, Brassicaceae 3%	*Fagopyrum esculentum* 2%, undetermined 1%, *Trifolium* spp. 1%<br>Asteraceae (*Helinathus* form) 1%, Asteraceae (*Taraxacum* form) 1%
HBH 10	*I. glandulifera* 85%	-	*Solidago* spp. 4%, *Frangula alnus* 3%	Asteraceae (*Helinathus* form) 2%, undetermined 2%, Brassicaceae 2%, *Fagopyrum esculentum* 1%, *Viola tricolor* 1%
